# The acute asymmetric effects of hemiovariectomy on testosterone secretion vary along the estrous cycle. The participation of the cholinergic system

**DOI:** 10.1186/1477-7827-4-11

**Published:** 2006-03-01

**Authors:** Angélica Flores, Jorge O Rodríguez, María T Palafox, Griselda Meléndez, Ana I Barco, Roberto Chavira, M Esther Cruz, Roberto Domínguez

**Affiliations:** 1 Biology of Reproduction Research Unit, FES Zaragoza, UNAM, México; 2 Instituto Nacional de Ciencias Médicas y de la Nutrición "Salvador Zubirán", México

## Abstract

The presence of asymmetry in the capacity of the left and right ovaries to secrete testosterone was analyzed by studying the effects of hemiovariectomy along the estrus cycle one hour after surgery. The effects of ether anesthesia on hormone serum levels were also analyzed.

Bilateral ovariectomy and the extirpation of the left ovary performed on the day of proestrus resulted in significantly lower testosterone levels.

Compared to the anesthetized group, the effects of perforating the peritoneum unilaterally varied according to the day of the estrous cycle and the side of the peritoneum surgery was performed on. Injecting atropine sulfate (ATR) to control or anesthetized rats on D1 resulted in a significant increase of testosterone serum levels.

The effects of perforating the peritoneum on testosterone levels depended on the cholinergic innervation and varied along the estrous cycle. Blocking the cholinergic system before performing unilateral or bilateral ovariectomy had different effects depending on the day of the estrous cycle. Testosterone plasma levels increased significantly when surgery was performed on the day of diestrus and dropped when surgery was performed on proestrus. Similar effects were observed when the left adrenal was extirpated from animals with the cholinergic system blocked. The results presented herein support the hypothesis of asymmetry in the ovaries' abilities to secrete steroid hormones, and that the capacity to secrete testosterone varies along the estrous cycle.

## Introduction

During the typical estrous cycle of common mono-ovulant animals, such as women, monkeys, cows, ewes, etc., the growth of a single dominant follicle is shown by only one of the ovaries, despite exposure to pituitary gonadotropins concentrations by perfusion of both ovaries with the same peripheral blood [1,2]. In the rat, a multi-ovulant animal, the left ovary releases more ova than the right one [3]. In gilts, the activities of cytochrome-c-oxidase, beta-N-acetyl-D-glucosaminidase, and glucose 6-phosphate dehydrogenase are quite dissimilar in the corpora lutea within the same ovaries, and in those from the right and the left ovary [4]. At present, no real explanation for these differences has been published, and it is possible that differences in ovarian innervation play a role on such event [2].

In previous studies we showed that performing acute hemiovariectomy to female cyclic rats on the day of estrus affects the concentrations in serum of progesterone, testosterone, and estradiol, and that these effects depend on which ovary, left or right, remains in-situ [5]. In the same study we showed that perforating the peritoneum unilaterally results in hormone serum levels changes, and that these changes depend on which side of the peritoneum the perforation is performed [5]. When the procedures were performed on diestrus 1, diestrus 2 or proestrus, the effects on progesterone serum levels depended on both, the ovary remaining in-situ and the day of the estrus cycle the animal was treated on. In addition, the results of the investigation established that the cholinergic system regulates progesterone release [6].

Testosterone is a hormone secreted by the ovaries and the adrenals [7] that serves as precursor for estradiol synthesis [7], and in several species plays a role regulating sexual behavior [8]. In women, testosterone production rates average 0.2 mg/day, with 25% secreted by the ovaries, 25% by the adrenals, and 50% arising from the peripheral metabolism of pre-hormones, notably androstenedione [9].

Previously, we showed that bilateral adrenalectomy and the extirpation of the left ovary on the day of estrus results in a significant decrease of testosterone serum levels, while extirpating the right ovary resulted in a significant increase in testosterone serum levels [5].

Since the ovaries' response to neuroendocrine control varies along the estrous cycle, the present study analyzed the acute effects on testostorone serum levels caused by performing unilateral ovariectomy on diestrus 1, diestrus 2, or proestrus. In addition, since the adrenals participate in establishing testosterone serum concentrations [5,10], the effects of unilateral and bilateral adrenalectomy on hormone ovarian secretion were analyzed, as well as the participation of the cholinergic system in the effects of unilateral and bilateral ovariectomy or adrenalectomized animals.

## Materials and methods

All experiments were carried out in strict accordance with the Guide for Care and Use of Laboratory Animals at the National Academy of Sciences. The Committee of the Facultad de Estudios Superiores Zaragoza approved the experimental protocols.

This study was performed with adult female rats from the CIIZ-V strain from our own stock (195-225-g body weight) that had shown at least two consecutive 4-day estrous cycles, monitored by cytological examination of daily vaginal smears. All animals were housed in an artificial light-dark cycle (lights on from 05:00 to 19:00 h), with free access to food (Purina S.A., México) and tap water ad libitum.

All surgeries were performed under ether anesthesia, between 13:00-13.15 h on diestrus 1 (D1), diestrus 2 (D2), or proestrus (P). Animals were sacrificed one hour after treatment, between 14.00-14.15 h.

Rats were randomly allotted to one of the experimental groups described below. Animals from different experimental groups were treated simultaneously. The distribution of animals used in each experimental group is presented in Tables 1, 2, 3 and 4.

**Table 1:** Means ± s.e.m. of testosterone serum concentration in control rats, and ether anesthesia treated animals, performed at 13.00 h on Diestrus 1, Diestrus 2 or Proestrus, sacrificed one hour later

**Group**	N	**Diestrus 1**	N	**Diestrus 2**	N	**Proestrus**
**Control**	7	6.3 ± 2.7	11	35.6 ± 6.9*	13	52.3 ± 10.2*
**Anesthesia**	7	14.0 ± 5.1	8	40.0 ± 11.0	8	116.0 ± 22.5^#^

*p < 0.05 vs. diestrus 1, MANOVA followed by Tukey's test

^#^p < 0.05 vs. control, Student's t test

**Table 2:** Means ± s.e.m. of testosterone serum concentration in control rats and animals injected with atropine sulfate (ATR) at 12.00 h on Diestrus 1, Diestrus 2 or Proestrus. The animals were sacrificed at 14.00 h.

**Group**	N	**Diestrus 1**	N	**Diestrus 2**	N	**Proestrus**
**Control**	7	6.3 ± 2.7	11	35.6 ± 6.9	13	52.3 ± 10.2
**ATR**	8	40.8 ± 6.4*	8	20.3 ± 3.4	8	89.4 ± 16.0
**ATR+Anesthesia**	10	30.3 ± 3.5*	8	23.6 ± 11.0	9	75.4 ± 8.3

*p < 0.05 vs. control group, MANOVA followed by Tukey's test.

**Table 3:** Means ± s.e.m. of testosterone serum concentration in rats submitted to bilateral perforation of the peritoneum (PPB), castration (CAS) or adrenalectomy (ADXB) on Diestrus 1, Diestrus 2 or Proestrus performed at 13.00 h, and animals submitted to the same treatments which were injected with ATR at 12.00 Animals were sacrificed at 14.00 h.

**Group**	N	**Diestrus 1**	N	**Diestrus 2**	N	**Proestrus**
**PPB**	8	8.6 ± 2.9	9	26.0 ± 6.5	9	206 ± 16.1
**ATR+PPB**	7	57.6 ± 9.1*	8	362.1 ± 53.7*	9	69.9 ± 15.8*
						
**CAS**	4	13.8 ± 5.3	4	5.0 ± 2.6	7	14.3 ± 2.9
**ATR+CAS**	8	30.3 ± 5.3*	8	19.9 ± 5.8*	10	<2.0*
						
**ADX B**	6	14.1 ± 4.4	7	33.9 ± 8.9	10	227.9 ± 19.3
**ATR+ADX B**	6	35.7 ± 8.9*	8	26.5 ± 5.0	7	80.8 ± 14.5*

* p < 0.05 vs. non-ATR treated group, Student's t test

### Experimental groups

#### Control group

Non-treated cyclic rats sacrificed at 14:00 h on D1, D2 and P.

#### Ether anesthesia

Groups of rats were anesthetized during 10 min on each day of the estrous cycle

#### Unilateral peritoneal perforation -sham surgery of unilateral ovariectomy or unilateral adrenalectomy- group

A unilateral incision was performed 2-cm below the last rib, affecting skin, muscle, and peritoneum. Neither ovaries nor adrenals were injured or manipulated. After surgical procedures the wound was sealed.

#### Bilateral peritoneal perforation -sham surgery of bilateral ovariectomy or bilateral adrenalectomy- group

A bilateral incision below the last rib, including skin and muscle, was performed. Neither ovaries nor adrenals were injured or manipulated. After surgical procedures the wound was sealed.

#### Unilateral ovariectomy

A unilateral incision below the last rib, including skin and muscle was performed, and the right or left ovary was extirpated. The wound was sealed after surgical procedures.

#### Unilateral adrenalectomy

A unilateral incision below the last rib, including skin and muscle, was performed and the right or left adrenal was extirpated. The wound sealed after surgical procedures.

#### Bilateral ovariectomy

A bilateral incision below the last rib, including skin and muscle was performed. The ovaries were removed, and the wound was sealed after surgical procedures.

#### Bilateral adrenalectomy

A bilateral incision below the last rib, including skin and muscle was performed. The adrenals were removed, and the wound was sealed after surgical procedures.

The effects of blocking the cholinergic system on testerone secretion by the ovaries, was analyzed by injecting atropine sulfate (ATR) (Sigma Chem. Co. St. Louis, Mo.) to additional groups of animals assigned to the same treatments as described above, one hour before surgery; at 12:00 h. ATR was injected at doses known to block ovulation: in D1, 100 mg/kg body weight (bw); in D2, 300 mg/kg bw; and in P, 700 mg/kg bw [11].

One hour after ATR treatment, groups of rats were randomly allotted to one of the following treatments: ether anesthesia, unilateral or bilateral peritoneal perforation, unilateral or bilateral ovariectomy or unilateral or bilateral adrenalectomy. All animals were sacrificed one hour after surgery.

Groups of untouched rats were injected with ATR on D1, D2 or P in the same dose as in each treatment group. The animals were sacrificed two hours after ATR treatment, between 14:00 - 14:15 h.

### Autopsy procedures

Rats were sacrificed by decapitation. The blood of the trunk was collected, allowed to clot at room temperature for 30 minutes, and centrifuged at 3,000 rpm during 15 minutes. Serum was stored at -20°C, until testosterone concentration was measured.

### Hormone assay

Concentrations of total testosterone in serum were measured by Radio-Immuno-Assay (RIA); using kits purchased from Diagnostic Products (Los Angeles, CA). Results are expressed in pg/ml. The Intra- and inter-assay variation coefficients were 5.6% and 8.7 %. The sensitivity of the assay was 0.2 pg/ml.

### Statistics

Data on hormonal concentrations in serum were analyzed using multivariate analysis of variance (MANOVA) followed by Tukey's test. Differences in serum hormone concentrations between two groups were analyzed by Student's t-test. A probability value of less than 5% was considered significant.

## Results

### Effects of ether anesthesia and unilateral perforation of the peritoneum

Rats from the untouched group sacrificed on D1 showed lower testosterone concentrations than animals sacrificed on D2 or P. Compared to their respective control group, ether anesthesia treatment on P resulted in significant increases of testosterone serum levels. Such effect was not observed when anesthesia treatment was performed on D1 or D2 (Table 1).

**Figure 1:**
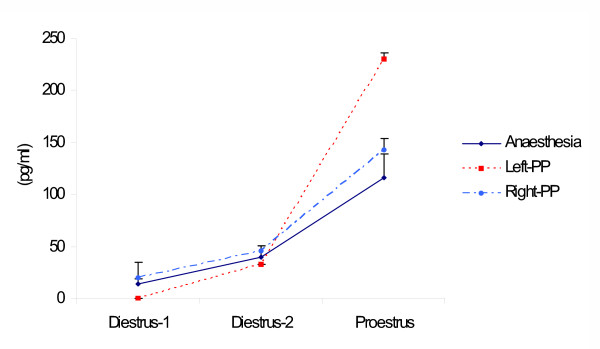
Means ± s.e.m. of testosterone serum concentration (pg/ml) in rats with left (LPP), right (RPP) or bilateral (BPP) sham operation performed at 13.00 h on Diestrus 1, Diestrus 2 or Proestrus, sacrificed one hour after surgery. * p < 0.05 vs. diestrus 1, MANOVA followed by Tukey's t test; * p < 0.05 vs. control, Student's t test.

Compared to the anesthetized group, the effects of unilaterally perforating the peritoneum varied according to the day of the estrous cycle and the side surgery was performed on. When the left side of the peritoneum was perforated on D1, testosterone serum levels were below the methodology's sensitivity (< 2 pg/ml); when performed on D2, no apparent effects were observed; but surgery performed on P resulted in a significant increase of testosterone levels. In turn, the unilateral perforation of the right side of the peritoneum did not significantly modify the concentration of testosterone (Figure 1).

### Effects of unilateral ovariectomy or adrenalectomy

**Figure 2:**
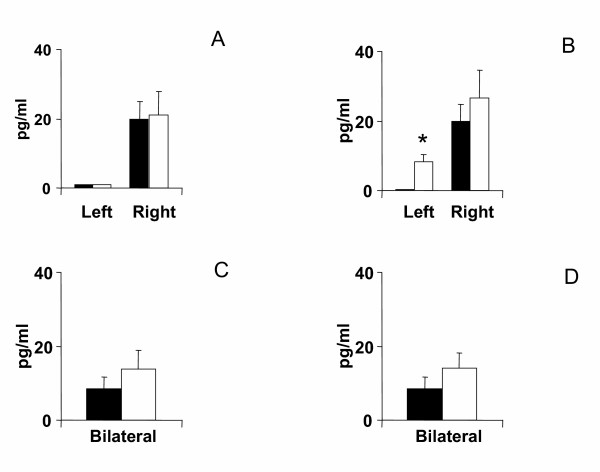
Effects of unilateral peritoneum perforation (panels A and B) black, unilateral ovariectomy (panel B) white, unilateral adrenalectomy (panel C) black, bilateral ovariectomy + left ovary extirpated (white), and (panel D) bilateral adrenalectomy + right ovary extirpated (white) performed at 13.00 h of the day of diestrus 1, on testosterone serum levels, measured 1 hour after surgery. * p < 0.05 vs. unilateral peritoneum perforation, Student's t test.

**Figure 3:**
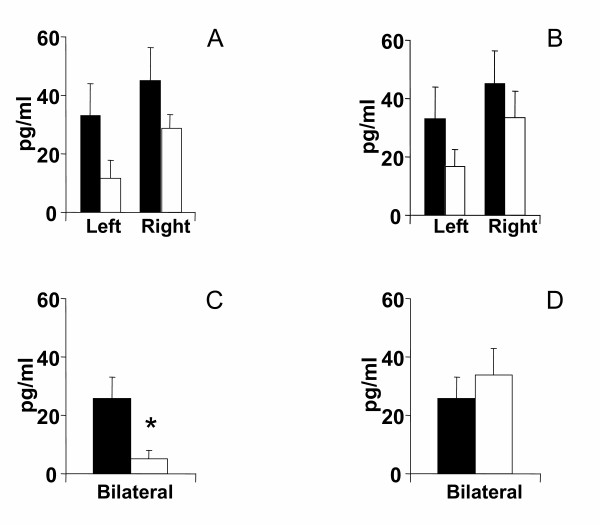
Effects of unilateral peritoneum perforation (panels A and B, black) unilateral ovariectomy (panel B, white), unilateral adrenalectomy (panel C, white) bilateral ovariectomy + left ovary extirpated and (panel D, white) bilateral adrenalectomy + right ovary extirpated performed at 13.00 h of the day of diestrus 2, on testosterone serum levels, measured 1 hour after surgery. * p < 0.05 vs. unilateral peritoneum perforation, Student's t test.

**Figure 4:**
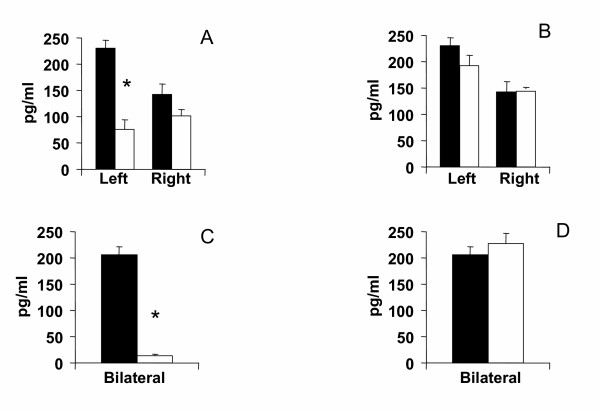
Effects of unilateral peritoneum perforation (panels A and B, black) unilateral ovariectomy (panel B, white), unilateral adrenalectomy (panel C, white) bilateral ovariectomy + left ovary extirpated and (panel D, white) bilateral adrenalectomy + right ovary extirpated performed at 13.00 h of the day of proestrus, on testosterone serum levels, measured 1 hour after surgery. * p < 0.05 vs. unilateral peritoneum perforation, Student's t test.

Compared to animals with a unilateral left peritoneal perforation, testosterone serum levels decreased significantly when left hemiovariectomy (right ovary in-situ) was performed on P. Left hemiovariectomy on D1 or D2 did not modify testosterone serum levels (Figures 2, 3, 4).

Regardless of the estrous cycle day when surgery was performed, right hemiovariectomy (left ovary in-situ) did not modify hormone serum levels (Figures 2, 3, 4). Compared to left peritoneal perforation, testosterone serum levels increased significantly when the left adrenal was extirpated on D1 (Figure 2). Extirpating the left adrenal on D2 or P did not modify testosterone serum levels (Figures 3, 4). In turn, regardless of the estrous cycle day when surgery was performed, extirpating the right adrenal did not modify testosterone serum levels (Figures 2, 3, 4).

### Effects of bilateral adrenalectomy or ovariectomy

Compared to the anesthetized group, the effects of bilateral peritoneum perforation performed on P resulted in increases of testosterone serum levels. In turn, the concentration of testosterone was not altered when surgery was performed on D1 or D2 (Figure 1).

Testosterone serum levels in bilaterally ovariectomized rats, studied on D2 or P, were lower than in counterpart animals with a bilateral peritoneum perforation. Castration had no apparent effect on testosterone levels when performed on D1 (Figures 2, 3, 4). Compared to animals with a bilateral sham operation, bilateral adrenalectomy did not modify testosterone serum levels (Figures 2, 3, 4).

### Effects of blocking the cholinergic system

Injecting ATR to control group rats in D1 resulted in a significant increase of testosterone serum levels. The same treatment performed on D2 or P resulted in non-significant changes (Table 2). Compared to ATR treated rats, anesthesia applied to ATR treated animals did not modify testosterone serum levels (Table 2).

**Figure 5:**
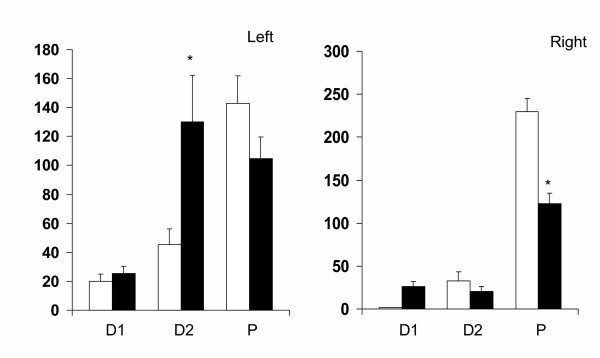
Comparative effects of atropine sulfate injection (black columns) to rats with unilateral peritoneal perforation (white) performed on diestrus 1 (D1), diestrus 2 (D2) or proestrus (P). * p < 0.05 vs. unilateral peritoneum perforation, Student's t test.

Injecting ATR to rats with a right peritoneal perforation performed on D2 resulted in a significant increase of testosterone serum levels. The same treatment on D1 or P did not modify testosterone serum levels (Figure 5). Blocking the cholinergic system, on the other hand, resulted in an increase of testosterone serum levels when the left peritoneal perforation was performed on D1. The same treatment performed to rats on P resulted in a significant decrease of testosterone secretion, while animals treated on D2 did not show apparent changes in testosterone serum levels (Figure 5).

#### Animals with the left organ (ovary or adrenal) in-situ

**Figure 6:**
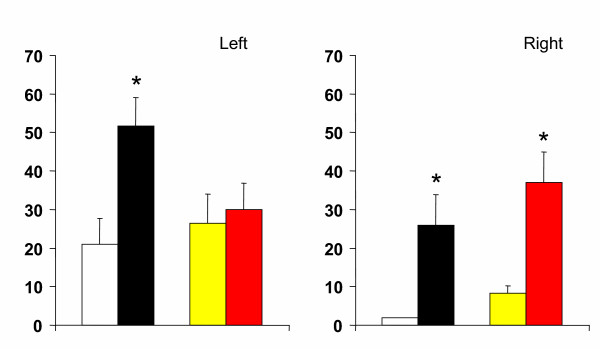
Comparative effects of atropine sulfate injection (black, red) to rats with unilateral ovariectomy (white) or unilateral adrenalectomy (yellow) performed on diestrus 1 sacrificed one hour after surgery. * p < 0.05 vs. unilateral peritoneum perforation, Student's t test

**Figure 7:**
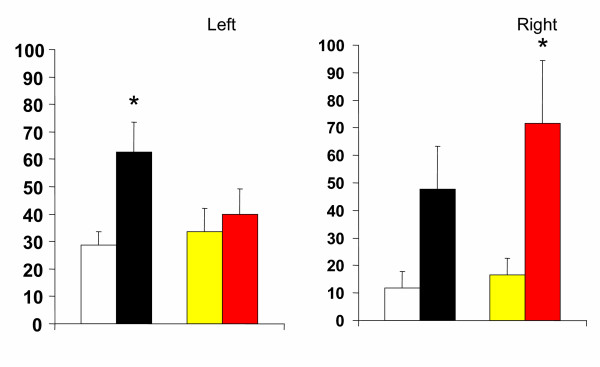
Comparative effects of atropine sulfate injection (black, red) to rats with unilateral ovariectomy (white) or unilateral adrenalectomy (yellow) performed on diestrus 2 sacrificed one hour after surgery. * p < 0.05 vs. unilateral peritoneum perforation, Student's t test

**Figure 8:**
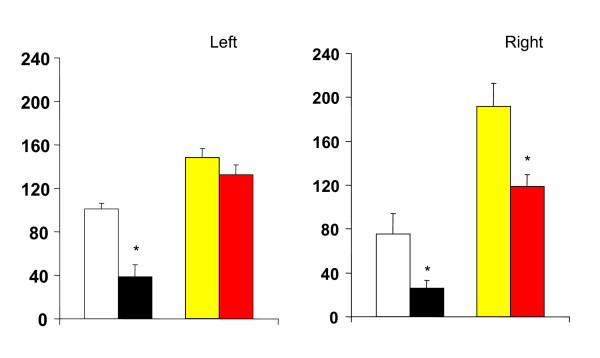
Comparative effects of atropine sulfate injection (black, red) to rats with unilateral ovariectomy (white) or unilateral adrenalectomy (yellow) performed on proestrus sacrificed one hour after surgery. * p < 0.05 vs. unilateral peritoneum perforation, Student's t test.

When the right ovary of ATR treated rats was extirpated in D1 or D2, testosterone serum levels were higher than in animals that did not have their cholinergic system blocked; while animals treated with ATR on P showed significantly lower testosterone serum levels. Non-significant changes in testosterone serum levels were observed in ATR-injected rats with the right adrenal extirpated (Figures 6, 7, 8).

#### Animals with the right organ (ovary or adrenal) in-situ

ATR-treatment resulted in similar hormone level changes as those observed in animals with the left ovary in situ, i.e. higher testosterone serum levels in rats treated on D1 or D2, and lower hormone levels in animals treated on P (Figures 6, 7, 8). When ATR-injected rats had the left adrenal extirpated on D1 or D2, a significant increase in testosterone serum levels was observed, while a significant decrease in testosterone levels was observed in rats treated on P (Figures 6, 7, 8).

#### Bilateral ovariectomized or adrenalectomized animals

Injecting ATR to rats with bilateral peritoneal perforation performed on D1 or D2 resulted in a significant increase of testosterone serum levels. The same treatment performed on P resulted in lower testosterone serum levels than in animals without ATR treatment (Table 3).

In bilaterally ovariectomized rats, blocking the cholinergic system on D1 or D2 resulted in higher testosterone serum levels than those observed in castrated rats (both ovaries removed). ATR treatment performed on P resulted in a significant drop of testosterone concentration, below the methodology's sensitivity. In bilaterally adrenalectomized animals, ATR treatment on D1 resulted in higher testosterone serum levels, while ATR treatment performed on P resulted in lower testosterone serum levels. No changes were observed when the treatment was performed on D2 (Table 3).

## Discussion

The results presented herein support the hypothesis of asymmetry in the ovaries' abilities to secrete testosterone [[5,6] and present results] and suggest that this ability varies along the estrous cycle.

In females, the sources of testosterone secretion vary with the species studied. In non-virgin female musk shrews, the adrenals are an important source of testosterone secretion and significantly contribute toward their hormonal control and sexual behavior of these [8]

Some questions arising from present results include: If ATR treatment blocks the surge of LH, and was applied at doses known to have such effect. Why wasn't such effect apparent in control animals treated with ATR on P and why would an effect be seen only on D1?

A possible explanation for the discrepancy in testosterone serum levels observed is that in D1, ATR treatment blocked testosterone conversion in estradiol, and its normal conversion in proestrus-treated ones. According to Cruz et al. [12], injecting ATR to rats on D1 results in a decrease in estradiol serum levels, while the same treatment performed had non significant effect in estradiol serum levels when performed on proestrus. Such possibility needs further study.

According to Labrie et al. [13,14], testosterone and estradiol can be produced by a large series of peripheral tissues containing the enzymatic systems required for the formation of active androgens and estrogens; and from a relatively large supply of precursor steroids; namely, dehydroepiandrosterone (DHEA) and its sulfate (DHEA-S) provided by the adrenals.

According to Sthal et al. [10], in female rats the gonads appear to be the main sources of testosterone, while the contribution of the adrenals to plasma testosterone levels is only moderate and relatively much smaller than in human beings. Since bilateral adrenalectomy did not modify them testosterone serum levels, present results agree with the idea that in the rat the adrenals do not contribute to testosterone serum levels. The results also support the idea that the ovaries are the main source of testosterone on the days of D2 and P, when bilateral ovariectomy resulted in a significant decrease of testosterone serum levels. However, since bilateral ovariectomy performed on the day of D1 did not modify testosterone serum levels, we postulate that most of the circulating testosterone on the day of D1 arises from other sources than the ovaries and adrenals. Klein et al. [15] proposed the existence of a viscero-visceral reflex mechanism that serves as a mechanism to modulate the activity of the principal ganglion cell in the sympathetic ganglion; and ultimately, the functional activity of specific visceral organs. The fact that both bilateral ovariectomy and adrenalectomy performed on D1 did not yield significant changes in testosterone serum levels could be analyzed under Klein et al. [15] idea. We propose that the presence of the adrenals in castrated animals compensate the lack of testosterone produced by the ovaries, and that the same mechanism is working in adrenalectomized rats. Because the ovaries and the adrenals receive neural inputs from the celiac ganglion, we propose that the changes in testosterone serum levels observed reflect some kind of neural information exchange between the ovaries and the adrenals.

Ether anesthesia activates the hypothalamus-pituitary-adrenal axis and the release of corticotopin releasing factor (CRF) by the hypothalamus, ACTH and β-endorphin by the pituitary, and corticosterone, norepinephrine, and epinephrine by the adrenals [16]. The results presented herein suggest that stimulating the hypothalamus-pituitary-adrenal axis with ether anesthesia on the day of P results in a significant increase in testosterone and progesterone serum levels [6]. Since progesterone is a precursor of testosterone, we propose that both increases are related to the stimulation of the hypothalamus-pituitary-adrenal axis provoked by ether anesthesia. However, the conversion of progesterone to testosterone did not occur when ether anesthesia was applied to rats in D1 or D2. In these animals, an increase in progesterone serum levels was observed [6], but testosterone serum levels were similar to control group levels. At present we do not have an explanation for such differences.

The superimposition of other stressors, such as surgery and peritoneum perforation, did not result in further increases of progesterone secretion [6]. However, the effects of perforating the peritoneum on testosterone serum levels depended on the side of the peritoneum perforated and the day of the estrous cycle when surgery was performed. According to Stener-Victorin et al [17], the bilateral electro acupuncture stimulation in the somatic segments of the ovaries' innervations results in an increase in neural growth factor levels in the ovaries, which in turn stimulates testosterone secretion.

The increase in testosterone secretion in response to an increase in neural growth factor levels has also been shown by Lara's group [18]. Since perforating the peritoneum affected the same somatic segments used by Stener-Victorin et. al. [17], we presume that bilateral and left side surgical procedures resulted in an increase of neural growth factor concentrations at the ovarian level, which in turn induced the increase in testosterone secretion. Perforating the right side of the peritoneum did not modify testosterone serum levels, while perforating the left side resulted in either a decrease (D1) or increase (proestrus) of testosterone serum levels, results that support our previous proposal that neural mechanisms regulate testosterone secretion by the ovaries [2].

Present results suggest that leaving the left organs (ovary or adrenal) in situ results in similar testosterone serum levels to those observed in rats with a unilateral perforation of the peritoneum. In turn, the right ovary does not have the same capacity to maintain testosterone serum levels. Such results support the idea that the degree pg endocrine asymmetry in the ovaries [[2], and present results] and the adrenals [present results] varies along the estrous cycle and depends on ovarian innervation.

According to Favaretto et al. [19], studying the effects of atropine on cultures of Leydig cells' ability to secrete testosterone showed that the parasympathetic autonomic system is involved in the inhibitory regulation of testicular androgen secretion. On the other hand, results by Wanderley et al. [20], and Chiocchio et al. [21], have shown that the sympathetic system stimulates testosterone secretion by Leydig cells. Because injecting atropine sulphate blocks the release of LH and ovulation [11,22], the increase in testosterone serum levels observed in rats injected with ATR on D1 can be interpreted as an effect of blocking the cholinergic system of the ovaries. Acetylcholine in the ovaries is provided by nerves and by its synthesis de novo by granulosa cells [23]. The fact that such effects were not observed in rats treated on D2 or P support the idea that the mechanisms modulating hormone release varies along the estrous cycle. Such interpretation is supported by the changes induced by injecting ATR to castrated and adrenalectomized rats, which varied depending on the day of the cycle when treatment was performed [present results].

The fact that the systemic blockade of the cholinergic system modified the effects of unilateral ovariectomy and not those of unilateral adrenalectomy could imply that the cholinergic innervation arriving to the ovary plays an inhibitory role on testosterone secretion.

Because testosterone can be produced by organs other than the ovaries and adrenals, and based on Labrie et al. [13,14] results, it is possible that the cholinergic system is involved in regulating testosterone synthesis. Another possibility is that the catabolism of testosterone is affected by the blockade of the cholinergic system in peripheral tissues, i.e. the liver.

There is evidence that acetylcholine (ACh) is a non-neuronal intra-ovarian signaling molecule, produced by granulosa cells (GCs), and that appears to act as signaling factor in the growing follicle. The ACh biosynthesis enzyme, choline-acetyltransferase (ChAT), is only expressed in growing antral follicles of rodent and primate species [23,24] By immunohystochemical and radioimmuno analysis, it has been shown that the granulose cells in the antral follicles synthesize acetylcholine [24]. The existence of M_1 _and M_5 _muscarinic receptors in granulosa cells from human and rat ovaries has been shown [24]. Also, the stimulation of cholinergic system in cow granulosa cells results in steroids synthesis increase [25]. To our knowledge no information on the effects of ATR on Ach release by granulose cells has been published, but we cannot discard that ATR is acting directly on the follicle, modifying the effects OF Ach in the follicle.

## Conclusion

Taken together, present results suggest that the regulation of testosterone secretion by the ovaries is a multifunctional process, and that ovarian innervation plays a key role.

The increase in testosterone serum levels observed in rats subjected to dorsal peritoneal perforation during proestrus suggest that during coitus, the mounts may stimulate the somatic segments associated with the innervation of the ovaries, resulting in an increase in testosterone levels female receptivity. Such increase in receptivity cannot be attributed to estrogens, since the estrogen serum levels in such rats was below than control group [12].
